# Inhibition of cyclooxygenase-2 decreases breast cancer cell motility, invasion and matrix metalloproteinase expression

**DOI:** 10.1186/1471-2407-6-181

**Published:** 2006-07-10

**Authors:** Teri L Larkins, Marchele Nowell, Shailesh Singh, Gary L Sanford

**Affiliations:** 1 Department of Microbiology, Immunology and Biochemistry Morehouse School of Medicine Atlanta, GA 30310-1495, USA; 2 Georgia State University Atlanta, GA 30302-3965, USA; 3 James Graham Brown Cancer Center University of Louisville, School of Medicine Louisville, KY 40202-1756, USA

## Abstract

**Background:**

Cyclooxygenase (COX) is the rate-limiting enzyme that catalyzes the formation of prostaglandins. The inducible isoform of COX (COX-2) is highly expressed in aggressive metastatic breast cancers and may play a critical role in cancer progression (i.e. growth and metastasis). However, the exact mechanism(s) for  COX-2-enhanced metastasis has yet to be clearly defined. It is well established that one of the direct results of COX-2 action is increased prostaglandin production, especially prostaglandin E_2 _(PGE_2_). Here, we correlate the inhibition of COX-2 activity with decreased breast cancer cell proliferation, migration, invasion and matrix metalloproteinase (MMP) expression.

**Methods:**

Breast cancer cells (Hs578T, MDA-MB-231 and MCF-7) were treated with selective COX-2 inhibitors (NS-398 and Niflumic acid, NA). Cell proliferation was measured by staining with erythrosin B and counting the viable cells using a hemacytometer. Cell migration and invasion were measured using migration and invasion chamber systems. MMP expression was determined by enzyme immunoassay (secreted protein) and real-time quantitative polymerase chain reaction (mRNA).

**Results:**

Our results show that there is a decline in proliferation, migration and invasion by the Hs578T and MDA-MB-231 breast cancer cell lines in the presence of either low concentrations (1 μM or lower) NA or NS-398. We also report that MMP mRNA and protein expression by Hs578T cells is inhibited by NS-398; there was a 50% decrease by 100 μM NS-398. PGE_2 _completely reversed the inhibitory effect of NS-398 on MMP mRNA expression.

**Conclusion:**

Our data suggests that COX-2-dependent activity is a necessary component for cellular and molecular mechanisms of breast cancer cell motility and invasion. COX-2 activity also modulates the expression of MMPs, which may be a part of the molecular mechanism by which COX-2 promotes cell invasion and migration. The studies suggest that COX-2 assists in determining and defining the metastatic signaling pathways that promote the breast cancer progression to metastasis.

## Background

Numerous studies indicate that cyclooxygenase-2 (COX-2) is highly expressed in a variety of human cancers, including colorectal, breast and prostate. In breast cancer, the expression of the COX-2 gene is associated with high tumor grade [1], which suggests it may serve as a prognostic biomarker for the presence of breast cancer. Researchers also found high expression of COX-2 in highly invasive estrogen independent breast cancer cell lines, (MDA-MB-231 (MDA-231) and Hs578T) as well as 12, 0-tetradecanoylphorbol-13-acetate (TPA)-induced COX-2 expression, while a poorly invasive and estrogen dependent cell line (MCF-7) did not express COX-2 [2,2,3]. Ristamaki *et al*. [4] also confirmed that the elevated COX-2 expression seen in 37.4% of the 1567 invasive breast cancers were associated with a large tumor size, high tumor grade, negative estrogen receptor status, high p53 expression and unfavorable prognosis. Transgenic mice that overexpressed COX-2 in mammary epithelial cells promoted mammary gland tumorigenesis and decreased apoptosis by reducing the expression levels of proapoptotic genes [4,5]. When transfecting the breast cancer cell line, MDA-MB-435 with COX-2, the cells migrated significantly better than the untransfected control cells [6]. The expression of COX-2 in breast tumors can be correlated with high metastatic potential.

Many of the critical steps of malignant tumorigenesis, such as cell proliferation, evading apoptosis, stimulating angiogenesis, enhancing cell motility, cell invasiveness and mediating immune suppression, have been associated with cyclooxygenase-2 expression. The end-products of COX-2 activity are prostaglandins and thromboxanes which may mediate these changes in cancer cell progression.

Elevated levels of prostaglandins, notably PGE_2_, have been detected in breast cancer cell lines, as well as invasive breast cancer [3,7,8]. Gilhooly *et al*. [2] induced COX-2 expression and activity in breast cancer cell lines with TPA which increased the production of PGE_2_. PGE_2 _was shown to stimulate cell proliferation indirectly by increasing estrogen levels via the induction of the aromatase gene expression [9]. Other researchers have shown that PGE_2_, prostacyclin and thromboxanes A_2 _contribute to tumor angiogenesis by mediating endothelial cell migration through integrin αVβ3 and by aiding in the production of angiogenic growth factors [10,11].

Recent data suggest a correlation between COX-2 expression and cell invasiveness. In order for cancer cells to metastasize, the cells must digest and dissolve the extracellular matrix (ECM) and the basement membrane, which requires the secretion and activation of MMPs. The expression and activation of MMPs may be directly proportional to the overexpression of COX-2 in tumor cells. One group has shown that Hs578T breast cancer cells transfected with COX-2 resulted in the activation of MMP-2 [12]. Sivula *et al*. [13] found increased COX-2 expression in breast cancer specimens, which also exhibited elevated MMP-2 expression and decreased disease specific survival. MMP-2 was elevated in 56 out of 59 invasive breast carcinomas in which expression of COX-2 was moderate to high. Studies also suggest that COX-2 may mediate urokinase plasminogen activator (uPA) production in metastatic breast cancer cell lines that overexpress COX-2. The uPA activates proteases and MMPs that degrade the basement membrane and mediate cytoskeleton reorganization. [6,12,14].

To our knowledge, we are the first to report evidence that COX-2 activity and expression may modulate the expression and activity of several MMPs in COX-2 expressing breast cancer cells. In this study, we screened for eight MMPs in breast cancer cells that were treated with and without of a COX-2 inhibitor. To date, only three groups have reported on studies focused only on the effect of COX-2 activity on the secretion of the gelatinases (MMP-2 and -9); all were done on cancers other than breast. Attiga *et al*. [15] have reported the inhibition of MMP-2 and MMP-9 by COX-2 inhibitors in prostate cancer. Tsuji *et al*. [16] observed an increase in MMP-2 activation and increase in MMP-14 mRNA expression by Caco-2 colon cancer cells, which showed high levels of COX-2. MMP-2 levels were decreased in the non-small cell lung cancer cell lines, A549 and H157 when treated with a COX-2 specific inhibitor [17].

The results of a number of epidemiological, clinical and laboratory studies suggest that the administration of COX inhibitors (NSAIDs, aspirin, indomethacin) reduces the incidence of breast, colon and prostate cancers [15,18-23]. Although several researchers have reported on the association of COX-2 overexpression with tumorigenesis in various cancers, many do not address the mechanism by which COX-2 promotes tumorigenesis. Using COX-2 inhibitors to prevent tumorigenesis will allow us to study the properties of COX-2 that influence breast cancer metastasis. The aim of the study reported here was to further elucidate the mechanism in which COX-2 promotes metastasis by using COX-2 selective inhibitors to study the role of COX-2 breast cancer motility and invasion. We were able to demonstrate that low and achievable concentrations of specific COX-2 inhibitors were sufficient to reduce the proliferation, migration and invasion of COX-2 expressing breast cancer cells. This study also suggests that COX-2 modulates the expression and activity of multiple MMPs involved in breast cancer metastasis.

## Methods

### Cell lines and cell culture

**Figure 1:**
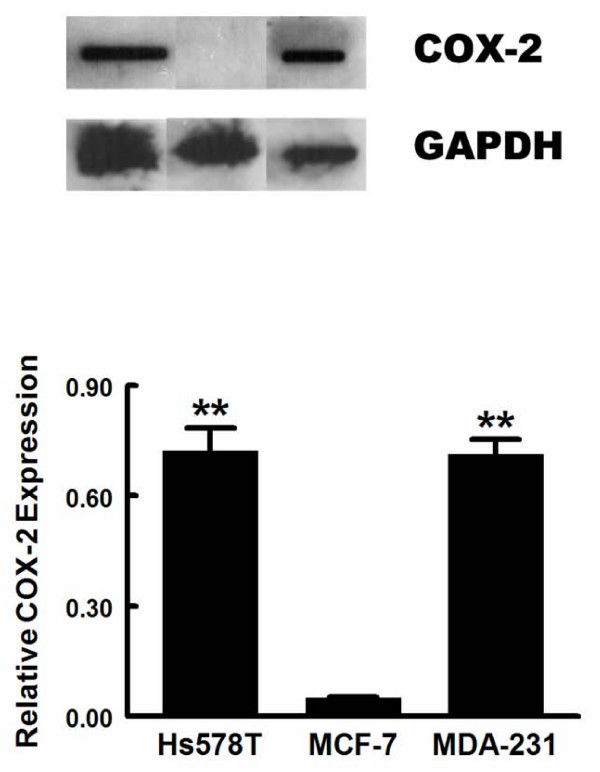
Expression of COX-2 protein in different breast cancer cell lines. (Hs578T, MCF-7 and MDA-231). Protein was extracted with RIPA buffer and the expression of COX-2 protein detected by immunoblotting and then quantitated by densitometry. Quantitated data were normalized to GAPDH. The relative units (RU) and ratio for the COX-2 and GAPDH bands for the different breast cancer cell lines were as follows: Hs578T (*COX-2*-57878/*GAPDH*-80182) = 0.7218, MCF-7 (*COX-2*-5871/*GAPDH*-74734) = 0.05, MDA-231 (*COX-2*-43861/*GAPDH*-61470) = 0.7135. Data for invasive cells lines were compared to poorly invasive cell line by one-way ANOVA followed by Student-Newman-Keuls test (,**p < 0.01).

The Hs578T, MDA-231 and MCF-7 breast cancer cell lines were all obtained from the American Type Culture Collection (Manassas, VA). The Hs578T and MDA-231 are estrogen-independent and highly invasive breast cancer cells. Although there have been studies that have looked at the expression of COX-2 in these breast cancer cell lines [2,3,24], we wanted to be certain that under our experimental conditions we saw similar results. We found high expression of COX-2 by both cell lines (Fig. 1). The MCF-7 cell line is an estrogen-dependent and poorly invasive breast cancer cell line that did not express COX-2. All cell lines were adapted for growth in Dulbecco's modified Eagle's medium (DMEM, Cambrex-Biowhittaker Walkersville, MD) supplemented with 0.1 Unit/ml bovine insulin (Sigma Chemical Co. St. Louis, MO), 100 I. U. penicillin, 100 μg/ml streptomyocin, 0.25 μg/ml amphotericin B (Cellgro Herndon, VA) and 10% fetal bovine serum (FBS Hyclone, Logan UT). When cells reached confluency, the cells were harvested by trypsinizing with 0.25% trypsin, 2.21 mM EDTA-Na in Hank's Balanced Salt solution w/Ca^2+^, Mg^2+ ^and NaHCO_3 _(Cellgro Herndon, VA), pelleted, resuspended in fresh medium and seeded in multiple 75-cm^2 ^flasks (Corning Corning, NY).

### Cell proliferation and viability

The Hs578T (1.5 × 10^5^), MDA-231 (1 × 10^4^) and MCF-7 cells (1.5 × 10^5^) were seeded on Falcon Multiwell™ 6-or 12-well plates (Becton Dickinson-BD Biosciences Discovery Labware Franklin Lakes, NJ) and grown for 48–72 h. The plates were washed with serum-free DMEM once and then incubated for 1 h in DMEM with 2% FBS. Control cells were treated with the vehicle only. Experimental cells were incubated with 0.1, 1.0 and 10 μM NS-398 (*N*-(2-cyclohexyloxy-4-nitrophenyl)-methanesulfonamide) or 10 μM niflumic acid (NA) for 24 h at 37°C in 5% CO_2_, harvested and homogeneous cell suspension prepared and counted. The number of viable cells was determined by the dye exclusion method using erythrosin B and measured by hemacytometer counting.

### Migration assay

Cell migration assays were performed using a modification of the protocol described by Attiga *et al *[15]. The BD Falcon Cell Culture Insert System containing PET (polyethylene terephthalate) membranes with 8 μm pores (BD Biosciences Discovery Labware Franklin Lakes, NJ) was utilized in the assay. The Hs578T and MDA-231 cells were harvested and resuspended into serum-free medium containing NS-398 (0.1, 1.0 and 10 μM) or the vehicle. The upper chamber of the insert was filled with 500 μl of the cell and drug suspension (1 × 10^5 ^cells) and 1.5 ml of (NIH/3T3) fibroblast-conditioned medium (FCM) was added to the lower chamber. FCM served as the chemoattractant. The conditioned medium was collected from NIH 3T3 cells grown in serum-free DMEM after 24 h. The plate was incubated in a humidified environment at 37°C with 5% CO_2 _for 24 h. After incubation, the cells were removed from upper surface of the membrane by wiping with a moist cotton swab. The lower surface of the membrane (cells that migrated) was stained for 10 min with 0.5% crystal violet in 25% methanol, rinsed with distilled water to remove excess stain not absorbed by cells and air-dried overnight. Digital images of the stained cells were obtained prior to the extraction of dye. The crystal violet was then extracted with 900 μl of 0.1 M sodium citrate in 50% ethanol. The absorbance was measured at 585 nm (using Genova Life Science Analyser (Jenway Felsted, England) spectrophotometer).

### Wound migration assay

The Hs578T and MDA-231 cells (2.0 × 10^5^) were seeded into six-well plates and grown to 100% confluency. The confluent cells were carefully wounded with sterile polished pasteur pipet tips and any cellular debris was removed by washing with PBS. The wounded monolayers were then incubated in the presence of NA (1.0 and 100 μM) for 0, 5 and 24 h time periods and digitally photographed. The distance between the wound edges was measured using Adobe Photoshop 6.0.

### Invasion assay

Cell invasion of the breast cancer cells were assessed by using the BD BioCoat™ FluoroBlok™ Invasion System (BD Biosciences Franklin, NJ) and procedures were followed according to the manufacturer. Monolayer cells grown to 80% confluency and labeled *in situ *with 10 μg/ml of 1,1'-didodecyl-3,3,3',3'-tetramethylindocarbocyanine perchlorate (DiIC_12_(3)) DiI fluorophore lipophilic tracer (Molecular Probe, Invitrogen Carlsbad, CA) in medium for 1 h at 37°C. The FluoroBlok Invasion insert plate was rehydrated with warm PBS for 2 h at 37°C. Cells were harvested and resuspended in serum-free DMEM containing NS-398 (0.1, 1.0 and 10 μM) or the vehicle. NIH/3T3 fibroblast-conditioned medium (FCM) 750 μl was added to the lower chamber and the cell suspension (500 μl) was added to the upper chamber. The system was incubated 22–24 h at 37°C and the fluorescence of the cells that invaded was read directly with a GENios Pro fluorescence plate reader (Tecan, San Jose, CA) at excitation/emission wavelengths of 535/590 nm.

### RNA isolation and real-time PCR

Total RNA was extracted from the Hs578T cells treated with NS-398 and/or PGE_2 _(control cells received the vehicle only), using the RNeasy mini Kit (Qiagen Valencia, CA) according to the manufacturer's instructions. The RNA was eluted and resuspended in RNA secure (Ambion San Diego, CA). The concentration and purity of the RNA was determined by measuring the absorbance at 260 nm (A_260_) and determining the ratio of the readings at 260 nm and 280 nm (A_260_/A_280_). Complimentary DNA (cDNA) was prepared from 2 μg of RNA using Omniscript Reverse Transcriptase (Qiagen Valencia, CA), 2.5 μM random primers (Invitrogen Carlsbad, CA) and 0.5 U/μl RNase Inhibitor (Ambion Austin, TX) and incubated at 37°C for 1 h.

The cDNA generated from the reverse transcriptase reaction was amplified by real-time PCR using specific MMP-(1, 2, 3, 9, 10, 11, 13, 14) and 18S primers and the SYBR Green polymerase chain reaction master mix reagents. The 18S rRNA was used a as a standard. We performed the PCR reaction according to the previously published methods in Singh *et al *[25]. The master mix for each cDNA sample was composed of 5 μl of the diluted cDNA (20 μl cDNA + 80 μl of nuclease-free water Ambion Austin, TX), 15 μl H_2_O, 5 μl 10 ng/ml reverse and forward primers, and the 25 μl Q™SyBr Green Supermix (Bio-Rad Hercules, CA). The reaction was performed according to the following program: 10 min at 95°C for activating the polymerase, the 40 cycles of 15 s at denaturation temperature of 95°C, 1 min of annealing at 60°C and the reaction was held at 4°C. The relative MMP mRNA expression to the 18S rRNA copies was quantified by real-time PCR analysis using the Bio-Rad Icycler and software (Bio-Rad Hercules, CA).

### Pro and active metalloproteinase protein detection

Cells (1 × 10^5^) were seeded in 12-well plates and grown to 70–80% confluency. The plates were washed with serum-free DMEM once and then incubated for 1 h in 1 ml of DMEM with 2% FBS and either NS-398 or the vehicle. The pro and active gelatinases (MMP-2 and MMP-9), collagenases (MMP-1 and MMP-13), and stromelysins (MMP-3 and MMP-10) levels released in the media were measured using a Quantikine colorimetric and Fluorokine fluorometric enzyme immunoassay (EIA) kit (R&D Systems Minneapolis, MN) using the manufacturer's instructions. After 24 h, the media were collected in microcentrifuge tubes and centrifuged for 10 min at 10,000 g in order to remove particulates. The samples were stored frozen at -80°C prior to the assay. The samples and MMP standards were incubated on a pre-coated polyclonal or monoclonal MMP antibody 96 well-plate for 2 h on a shaker at room temperature. The intensity of color on the Quantikine EIA plates was determined by Spectra Max 190 and SOFTmax-Pro 4.3 Life Sciences Ed. (Molecular Devices Sunnyvale, CA) at a wavelength of 450 nm with a correction of 540 nm. The fluorescent signal in the sample on the Fluorokine EIA wells was determined by the GENios Pro fluorescence plate reader (Tecan, San Jose, CA) at excitation/emission wavelengths of 340 nm/465 nm.

### Statistical analysis

Statistical significance was determined by one-way ANOVA with Student-Newman-Keuls post test was performed using GraphPad InStat v3.00. The data was expressed as the mean ± S.E.; significance was achieved at p values < 0.05.

## Results

### Growth inhibition of COX-2 expressing breast cancer cells

**Figure 2:**
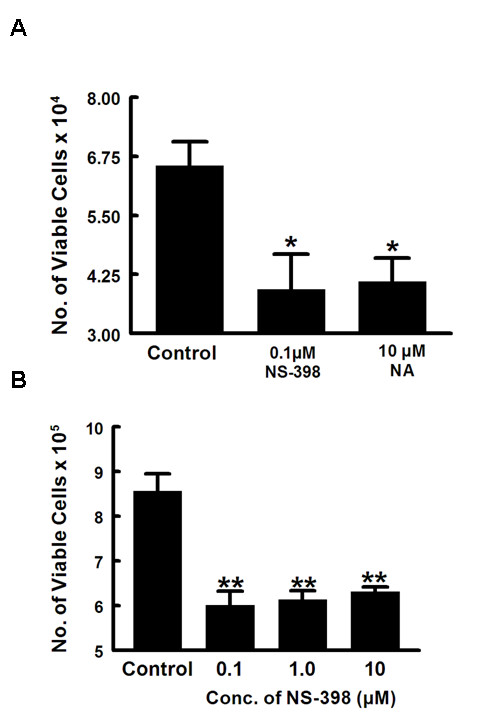
Effect of COX-2 inhibitors on breast cancer cell proliferation. **A**) 1 × 10^4 ^MDA-231 cells and **B**) 1.5 × 10^5 ^Hs578T were seeded, grown for 48 h and then treated with COX-2 inhibitors (NS-398 and Niflumic Acid) or the vehicle for 24 h. Cells were harvested and counted as described in the Methods. Data for treated cells were compared to untreated controls by one-way ANOVA followed by Student-Newman-Keuls test (*p < 0.05, **p < 0.01).

We treated the Hs578T, MDA-231 and MCF-7 breast cancer cells with NS-398 and NA for 24 h and examined the effects of the cell proliferation. Treatment of the two COX-2 expressing cell lines, Hs578T and MDA-231 with 0.1, 1.0, 10 μM NS-398 and NA impeded the growth of the cells. NS-398 resulted in a 30% growth inhibition of Hs578T cells (p < 0.01) and 40% growth inhibition of MDA-23l cells (p < 0.05) at 0.1 μM (Fig. 2). We also found a 37% inhibition (p < 0.05) of MDA-231 growth by 10 μM NA compared to the control (Fig. 2b). The MCF-7 cells did not show a difference in growth when treated with NS-398 (data not shown). The COX-2 inhibitors did not significantly affect the cell viability. There was 85%, or higher, cell viability seen after treatment with COX-2 inhibitors, up to 10 μM indicated no cytotoxicity.

### Inhibition of cell migration and invasion with a COX-2 inhibitor

**Figure 3:**
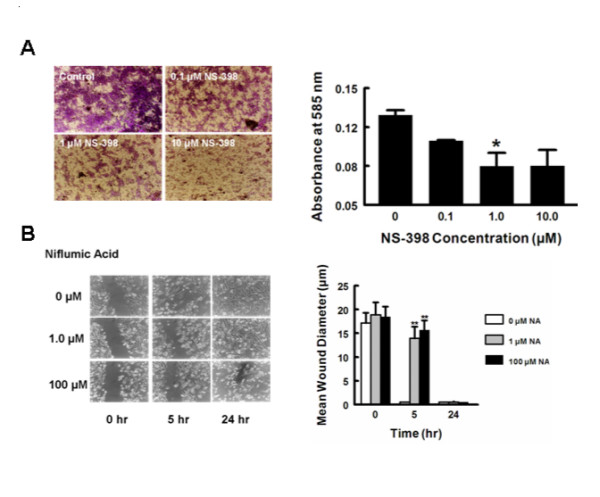
The effect of COX-2 inhibition on the migration of MDA-231 cells. **A**) Cells (1 × 10^5^) were placed in the upper chamber inserts with or without NS-398 in serum-free DMEM and allowed to migrate for 24 h. FCM was used as a chemoattractant to stimulate the migration of the cells. **B**) Wound migration assay. Confluent MDA-231 cells cultured in six well dishes were wounded with a sterile pipette tip and then incubated with or without Niflumic Acid for 24 h. Photographs were taken with a phase contrast microscope and measurements with Adobe photoshop 6.0. Values were significant (*p < 0.05, **p < 0.01) when compared to untreated controls (0 μM NA or NS-398).

We evaluated the effect of the COX-2 inhibitors on the cell motility and invasiveness of MDA-231 and Hs578T breast cancer cells. Cell motility and invasion are a measure of metastatic potential cancer cells. Treatment with 1.0 μM NS-398 inhibited MDA-231 cell motility by 35% (p < 0.05) (Fig. 3a). Confluent MDA-231 cells subjected to the scratch wound assay in the presence of 1.0 and 100 μM NA showed a significant delay in cells moving into the injury area. The control cells migrated into the wound area by 5 h to such an extent that the wound edges were indistinguishable, whereas the experimental group of cells did not migrate into and completely close the wound area until 24 h (Fig. 3b).

**Figure 4:**
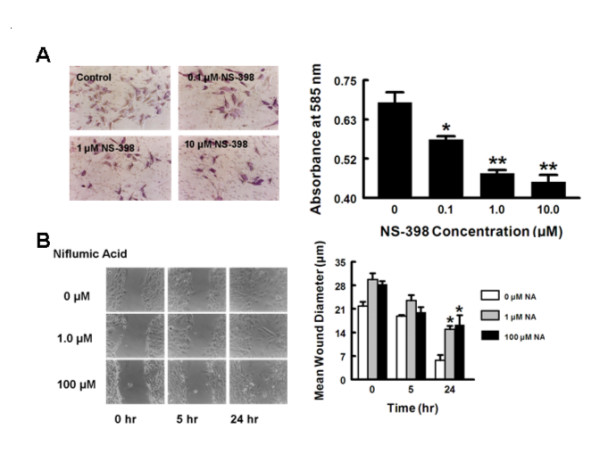
The effect of COX-2 inhibition on migration of Hs578T cells. **A**) Cells (1 × 10^5^) were placed in the upper chamber inserts with or without NS-398 in serum-free DMEM and allowed to migrate for 24 h. FCM was used as a chemoattractant to stimulate the migration of the cells. **B**) Wound migration assay. Confluent Hs578T cells cultured in six well dishes were wounded with a sterile pipette tip and then incubated with or without Niflumic Acid for 24 h. Photographs were taken with a phase contrast microscope and measurements with Adobe photoshop 6.0. Values were significant (*p < 0.05) when compared to untreated controls (0 μM NA or NS-398).

Figure 4a shows that the motility of Hs578T cells was significantly decreased by 21% (p < 0.05) by 10 μM NS-398 when compared to the control. Inhibition of Hs578T cell motility by COX-2 inhibitors was also confirmed by the scratch wound assay. Figure 4b shows that Hs578T wound incubation with 1 and 100 μM NA resulted in a significant delay in cell migration compared to the control (Fig. 4b).

**Figure 5:**
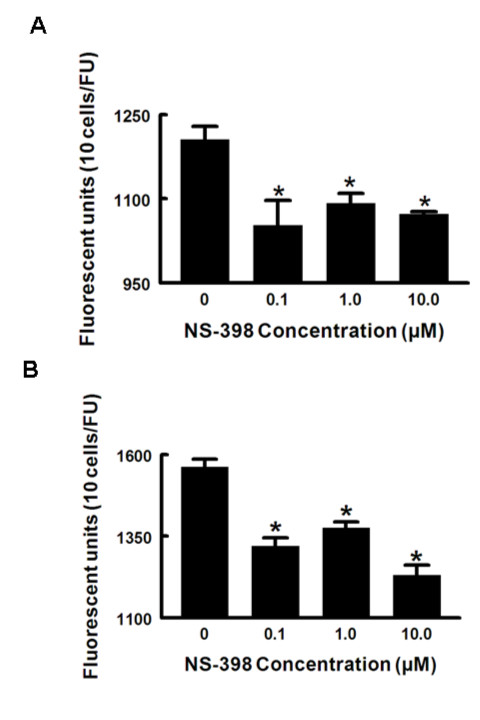
The effect of NS-398 on invasion of MDA-231 (A) and Hs578T (B) cells. Cells (1 × 10^5^) were placed in the upper chamber of the BD BioCoat Tumor Invasion FluoroBlok System with and without NS-398 in serum-free DMEM and allowed to invade for 24 h. FCM was used as a chemoattractant to stimulate the invasion of the cells. Values were significant (*p < 0.05, **p < 0.01) when compared to untreated controls (0 μM NS-398).

Prior to the migration phase of metastasis, breast cancer cells must invade the basement membrane and the extracellular matrix. We measured the invasive ability of breast cancer cells on Matrigel coated membranes. Invasion of MDA-231 cells was inhibited by 11% in response to 10 μM NS-398 (p < 0.01) (Fig. 5a). We found a 21% decrease of the invasion of Hs578T cells by 10 μM NS-398 (Fig. 5b).

### Effect of COX-2 inhibition on the secretion of MMPs

**Figure 6:**
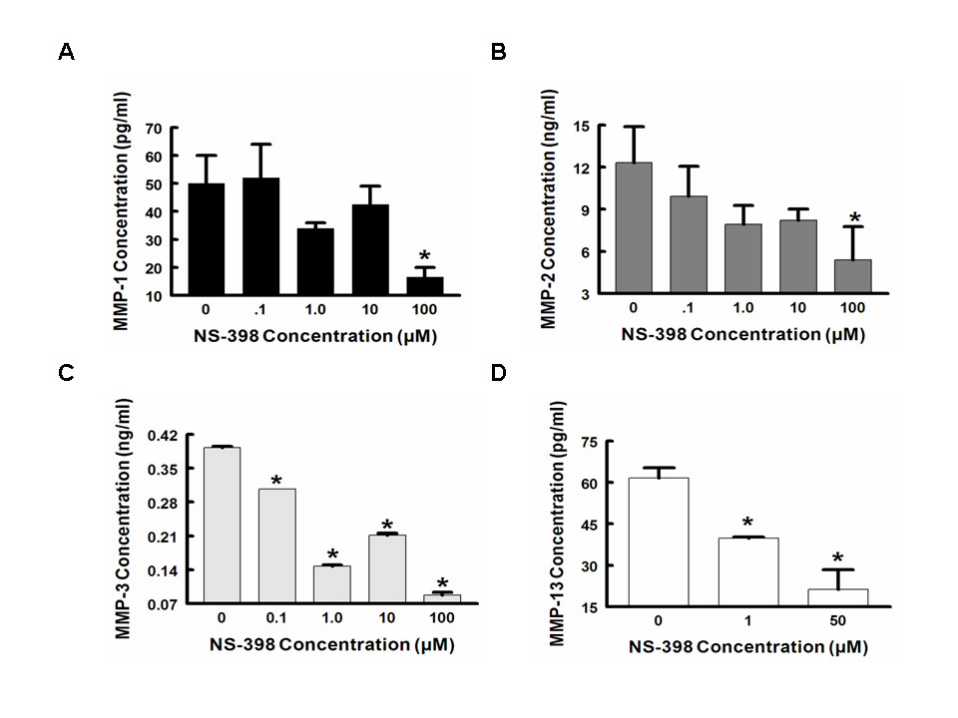
The effect of NS-398 on metalloproteinase release in culture media by Hs578T cells. The cells were cultured in the presence or absence of increasing doses of NS-398 for 24 h. The levels of the pro and active form of MMPs-1, 2, 3, 9, 10 and 13 were measured in the culture media of treated cells by EIA. **A**) MMP-1 **B**) MMP-2 **C**) MMP-3 **D**) MMP-13. MMP-9 and MMP-10 were not detected in Hs578T media. Values were significant (*p < 0.05) when compared to untreated controls (0 μM NS-398).

We assessed the dose-dependent effects of NS-398 on MMP secretion by Hs578T and MDA-231 cells. After 24 h, there was a 67% decrease in MMP-1, 45% in MMP-2 and 65% in MMP-3 secretion by Hs578T cells treated with 100 μM NS-398 (Fig. 6). We also observed a significant reduction (66%) of MMP-13 when the cells were treated with 50 μM NS-398 (Fig. 6d). In contrast, MMP-9 and MMP-10 were undetectable in the media of Hs578T cells, suggesting that the cells did not secrete these MMPs.

**Figure 7:**
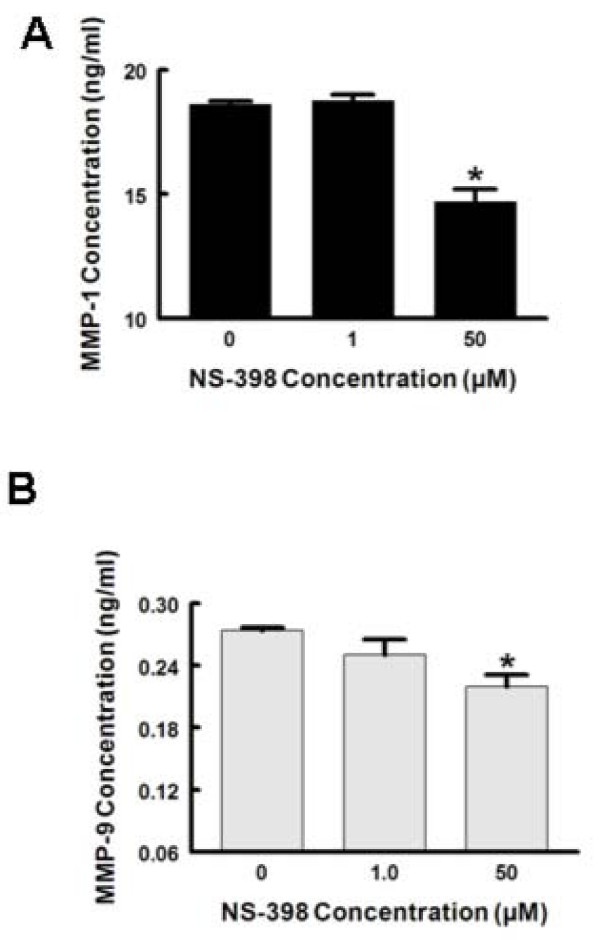
The effect of NS-398 on metalloproteinase release in culture media by MDA-231 cells. These cells were cultured in the presence or absence of increasing concentrations of NS-398 for 24 h. The secretion of the pro and active form of MMPs was determined by EIA. **A**) MMP-1 **B**) MMP-9. MMP-2, MMP-3, MMP-10 and MMP-13 were not detected in MDA-231 media. Values were significant (*p < 0.05) when compared to untreated controls (0 μM NS-398).

Out of the six MMPs assessed by EIA, we were only able to detect the presence of MMP-1 and MMP-9 in the media of MDA-231 cells. There was about a 20% decrease for both MMP-1 and MMP-9 secreted by these cells treated with 50 μM NS-398 (Fig. 7). We found a 15% reduction in active MMP-2 and 20–30% reduction in active MMP-9 by immunoblotting analysis for MDA-231 cells treated with the NA or NS-398, respectively (data not shown).

### MMP mRNA expression in Hs578T in the presence of COX-2 inhibitor

Table 1 shows detectable basal levels of MMPs (1, 2, 3, 9, 10, 11, 13 and 14) in the COX-2 expressing cell line, Hs578T before treatment with NS-398 with or without PGE_2_. MMP-2 and -3 exhibited the highest number of transcripts produced by the cells compared to the other MMPs at basal levels which corresponds with the trend of the active protein secreted by the Hs578T. The MMPs that displayed barely detectable transcripts at basal levels were MMP-10 and -14. This is consistent with EIA results, when active MMP-10 was not detected in the media. We observed a significant 2 log-fold and 4 log-fold decline in MMP-2 and MMP-3 expression levels when the cells were treated with 10 μM NS-398. MMP 1, 9, 11 and 14 were either below detection limits or at barely detectable levels when the Hs578T cells were treated with 10 and 100 μM NS-398. One of the products of COX-2 is PGE_2_; this prostaglandin reversed the inhibitory effect of NS-398 on the MMP mRNA expression. In some cases PGE_2 _actually resulted in MMP levels that were greater than control levels. These data suggests that NS-398 reduced MMP mRNA expression and that PGE_2 _may promote the invasion of breast cancer cells through enhancing MMP secretion.

## Discussion

Prognosis of breast cancer patients is strongly correlated with the stage of the cancer at the initial diagnosis. If the cancer is only detected when there is an invasive state, then the prognosis is poorer. In fact, 90% of patients that die of breast carcinoma have bone metastases [26]. COX-2 expression may serve as a biomarker that could be assessed to predict the possible progression of the disease. Additionally, understanding the mechanistic and molecular role of COX-2 in tumor progression and the complex multi-step metastatic process can aid in combating breast cancer mortality. In order for metastasis to occur, angiogenesis, cell attachment, dissociation, proteolysis of the matrix and motility are essential steps. Several studies have reported that COX-2 is involved in these complex steps. In this study, we examined the role of COX-2 in breast cancer cell proliferation, invasion and motility in an attempt to improve our understanding of the molecular mechanism of breast cancer metastasis.

Several studies have demonstrated the efficacy of non-selective and selective COX inhibitors on the proliferation of breast cancer cells, using murine cancer models and human cancer cell lines. Harris *et al*. [27] evaluated the chemopreventive potential of celecoxib and ibuprofen in the DMBA (7, 12-dimethyl-benz [a] anthracene) model of breast cancer in Sprague-Dawley rats. Both drugs significantly reduced tumor incidence, volume and burden. At 40 and 60 μM, celecoxib suppressed the growth and proliferation in breast cancer cell lines, MDA-231 and MDA-435 by inducing apoptosis and cell cycle arrest at the G_0_/G_1 _phase, respectively [28]. Other studies have suggested that high concentrations of COX inhibitors (over 25 μM) promote apoptosis in cancer cells [29-32]. Although we did not detect any effects that may be independent of COX inhibition, other groups have demonstrated that high concentrations of non-selective and selective COX-2 inhibitors may reduce the proliferation or modulate the cell cycle genes in cancer cells independent of COX-2 expression [33-35]. Elder *et al*. [34] found a dose-dependent anti-proliferative effect of NS-398 in the colorectal cancer cells, S/KS, that does not express detectable levels of COX-2. Both NS-398 and nimesulide induced p21 gene promoter activity in non-small cell lung cancer cells but COX-2 siRNA did not affect the expression of p21 [35]. Based on these prior studies using the selective COX-2 inhibitors, we selected a concentration range of 0.1–100 μM to use in our experimental studies. We found that the lower concentrations significantly affected the breast cancer cells to about the same extent as the higher concentrations [36-38]. The levels that we used are in the achievable range for humans that have been reported for clinically available selective COX-2 inhibitors used as drugs, e.g., celecoxib and meloxicam.

In this study, we demonstrated the anti-proliferative effects of the selective COX-2 inhibitors, NS-398 and NA in the both COX-2 expressing cell lines, MDA-231 and Hs578T. Our findings indicate that these selective inhibitors will only retard the growth of breast cancer cells that express COX-2. The inhibition of Hs578T cell growth reached a plateau around 1.0 μM indicating there would be no further decrease at higher concentrations. While a few studies may show that MCF-7 cell growth is inhibited by COX-2 inhibitors [39,40], we found that MCF-7 cancer cells were not affected by the COX-2 inhibitors (data not shown). The inhibition of cancer cell proliferation by COX-2 inhibitors has also been reported for prostate and colon cancer models [41-43].

Cell motility is one of the last critical steps of metastasis which is necessary for a cell to move through the extracellular matrix and enter the circulation where it can travel to a distant site. We were able to demonstrate the inhibitory effect of the selective inhibitors on the motility of the Hs578T and MDA-231 cells. Similarly, Singh *et al*. [6] showed the addition of 50 μM NS-398 inhibited MDA-231 cell migration by 47%. Our results also suggest even a lower concentration (i.e. 0.1 μM) of NS-398 causes a decline in the cell motility of the MDA-231 cells. This data confirms that COX-2 activity mediates the chemotaxis of breast cancer cells across a membrane toward a chemoattractant.

With Matrigel, we found that NS-398 partially attenuated the invasive ability of the COX-2-expressing cell lines. Compared to the control, 10 μM NS-398 reduced the invasion of both of the COX-2-expressing cell lines by 10–20%, which is consistent with the findings of Singh *et al *[6]. These researchers showed that the addition of a higher level of NS-398 (50 μM) inhibited MDA-231 invasion through Matrigel by 54%.

In order for the cells to invade and migrate through the basement membrane (i.e., Matrigel), proteolysis of the extracellular matrix must occur. This is accomplished by the secretion and activation of MMPs, which will degrade all extracellular matrix components (e.g., laminin, collagen (all types), entactin and a number of other factors, including growth factors (TGF-β and FGF) and cytokines). The association of MMPs with tumor progression is well documented [7,44,45]. The expression of MMPs, particularly the gelatinases (MMP-2 and MMP-9) have been associated with high potential of metastasis in several human carcinomas including breast cancer [46,47]. In this study, we also determined the mediation of MMP expression and secretion by COX-2, using an inhibitor approach.

We found a dose-dependent inhibitory effect of NS-398 on the secretion of both pro and active forms of MMPs (1, 2, 3, and 13) in Hs578T cells, which highly express COX-2. With 1 μM of NS-398 treatment, we found that MMP secretion levels decreased by 50% or higher. Surprisingly, we did not detect quantifiable levels of the pro and active MMP-9 which has been suggested to be highly expressed by invasive breast carcinomas [48]. We did observe very low MMP-9 mRNA expression in control Hs578T cells, which could account for these results. We were also able to detect an effect of COX-2 inhibition on the expression of MMP mRNAs. NS-398, 1 μM, decreased MMP mRNA expression to barely detectable levels; there was little change in expression with 10 or 100 μM NS-398. This suggest that either there is not dose-dependency or there is a threshold effect of NS-398 on MMP mRNA levels, i.e., a minimum level of COX-2 activity is required to induce the expression of MMP mRNA that we observed in control cells. The 1 μM concentration is in the range of the IC_50 _of NS-398 for the inhibition of COX-2 enzyme activity reported by the manufacturer and by previous studies [49,50], which may account for the drastic decrease it caused in MMP mRNA expression.

Increased levels of various prostaglandins, especially PGE_2_, have been associated with many different invasive cancers [7,51-53]. Our position is that the ability of NS-398 to produce an anti-invasive effect on cancer cell lines is due to the down-regulation of prostaglandin production, which facilitates the functions of COX-2. Our findings also show that COX-2 may exert a tumorigenic effect through PGE_2_. By inhibiting the COX-2 activity in the Hs578T cells with 1–10 μM NS-398, we were able to reduce the endogenous PGE_2 _levels by 75% (unpublished observations). The addition of exogenous PGE_2 _completely reversed the inhibitory effect of with NS-398 on the expression of all MMP mRNAs by Hs578T cells. In fact, PGE_2 _treatment of the NS-398 pre-treated Hs578T cells resulted in increased MMP expression, which surpassed basal levels. We also found that NS-398 treatment of MDA-231 also resulted in the inhibition of the pro and active MMPs (1, 2, and 9). Our preliminary studies showed that adding exogenous PGE_2 _to the control MDA-231 cells did not affect or may have decreased the secretion of MMPs. These data suggest that exogenous PGE_2_, by itself, can not modulate the increase in MMP secretion without a reduction in the endogenous PGE_2 _levels. Several other studies have also implicated PGE_2 _in the activation of COX-2 gene expression, which could lead to an increase in active COX-2 and subsequent higher MMP expression [54,55]. Pan *et al*. [56] showed that NS-398 treatment resulted in a suppression in MMP-2 promoter activity, MMP mRNA and active MMP-2 protein by the A549 lung cancer cell line. Inhibition of the MMP-2 promoter activity by NS-398 was partially reversed by exogenous PGE_2_. Other studies using a human prostate cancer cell line (DU-145) or a colorectal cancer cell line (MC-26), treated with 10 and 100 μM NS-398, reported a reduction in the release of pro and active MMP-2 and MMP-9 in the culture media [15,38]. However, the underlying mechanism for how PGE_2 _up-regulates the expression of MMPs is not known. Further investigations are needed to properly explain the phenomenon that we observed in our study.

Contrary to our expectation, we did not see exactly the same MMPs secreted by both of the COX-2-expressing cell lines. We suspect that the difference could be a result of the heterogeneity of the Hs578T cell line or the cell's response to its extracellular environment. The time period used for transcription of the various MMPs in these cells may also explain some of the variability observed. In an *in vivo *system, the stroma of breast carcinomas may also secrete MMPs in response to high COX-2 expression by the breast tumor, which would also aid in tumor cell invasion. To our knowledge, we are the first to report that the inhibition of COX-2 reduces both MMPs mRNA expression and secretion of pro and active MMPs in breast cancer cell lines where COX-2 is highly expressed. Our study suggests that the MMPs may promote some of the deleterious effects of COX-2, and could possibly be studied as a useful target for combination chemotherapy for breast cancer patients that overexpress COX-2.

## Conclusion

Using an inhibitory approach, we examined the involvement of COX-2 activity promoting breast cancer metastatic behavior. In this report, we confirm that the expression and activity of COX-2 may be a required component for breast cancer cell proliferation, motility and invasion. In our experiments, we showed that treating breast cancer cell lines that express COX-2 with a COX-2 inhibitor decreased proliferation, migration, invasion, and MMP production. Furthermore, we report that PGE_2 _may mediate the effects COX-2 activity by activating signaling pathways via PGE_2 _(EP) receptors. However, exogenous PGE_2 _alone was not able to induce a number of changes seen by exogenous PGE_2 _when COX-2 was first inhibited. Since currently there is no clear understanding of the mechanism by which COX-2 facilitates the progression of cancer, our studies suggest a strategy for assessing the COX-2 pathway through elucidating possible downstream signaling mediators that have effects on the migration, invasion and expression of MMPs by highly invasive breast cancer cell lines.

## Competing interests

The author(s) declare that they have no competing interests.

## Authors' contributions

TLL implemented the experimental design, carried out the assays and drafted the manuscript. MN participated in performing the cell proliferation studies and the western blotting analysis for MMP protein expression. SS designed and provided the MMP primers, 18S primers, 18S standard and the set-up for the real-time PCR. GLS aided in the experimental design for the studies, provided the majority of the laboratory materials/equipment and in drafting the manuscript.

## Pre-publication history

The pre-publication history for this paper can be accessed here:



## Figures and Tables

**Table 1:** Effect of COX-2 Inhibitor and PGE_2 _on MMP mRNA Expression. Expression of MMP mRNA in Hs578T cells in the presence of NS-398 with or without 1 μM PGE_2_. Hs578T cells were cultured in the presence NS-398 with or without PGE_2 _for 24 h. The relative quantitation of the gene expression of the MMP mRNA was calculated against an 18S rRNA standard. The number of copies of MMP mRNA per 10^6 ^copies of 18S rRNA was determined by real time-PCR according to the methods of Singh et al [25]. The real-time PCR analysis was capable of detecting >5 copies of mRNA. Hence, copy numbers <5 were below detection (BD) limits. Significance (n = 4, *p < 0.05) was determined by comparing the treated Hs578T cells to the untreated control. ^#^The data in this column has an n = 2 and is listed to show the direction of the effect of PGE_2_.

**Type of MMP**	**NS-398 (μM)**				**NS-398/PGE_2 _(μM) #**
	**Control**	**1**	**10**	**100**	**1.0/1.0**
**MMP-1**	1.04 × 10^2^	BD	BD	BD	3.58 × 10^3^
**MMP-2**	2.09 × 10^5^	3.93 × 10^3^*	3.87 × 10^2^*	1.06 × 10^3^*	3.11 × 10^7^
**MMP-3**	3.42 × 10^5^	5.73 × 10^2^*	28.0*	1.08 × 10^2^*	3.26 × 10^13^
**MMP-9**	16.4	BD	BD	BD	1.06 × 10^5^
**MMP-10**	BD	BD	BD	BD	38.4
**MMP-11**	8.31 × 10^3^	6.92	BD	BD	1.85 × 10^9^
**MMP-13**	3.93 × 10^2^	57.9	11.7	4.83	1.70 × 10^6^
**MMP-14**	BD	BD	BD	BD	4.53 × 10^4^
